# Use of Mineral Trioxide Aggregate in Surgical and Conventional Endodontics: A Report of Five Cases

**DOI:** 10.5005/jp-journals-10005-1206

**Published:** 2013-08-26

**Authors:** Seema Gupta, Mridula Goswami

**Affiliations:** Senior Resident, Department of Pediatric Dentistry, Maulana Azad Institute of Dental Sciences, New Delhi, India, e-mail: seema.mds@gmail.com; Professor and Head, Department of Pediatric and Preventive Dentistry Maulana Azad Institute of Dental Sciences, New Delhi, India

**Keywords:** Apexification, Calcium hydroxide, Mineral trioxide aggregate

## Abstract

Facial trauma that results in fractured, displaced or lost teeth can have significant negative functional, esthetic and psychological effects on children. An acute dental trauma may imply impact to the hard dental tissues and damage to the pulp and periodontium, ultimately leading to partial or total pulp necrosis and/or root resorption. Apexification is a commonly used procedure for treating and preserving immature permanent teeth that have lost pulp vitality. Immature teeth undergoing apexification were earlier filled with calcium hydroxide paste for the purpose of disinfection and induction of an apical calcific barrier. However, certain drawbacks led to the use of mineral trioxide aggregate (MTA) to fill the apical end without the need for calcific barrier formation. This article demonstrates the use of MTA as an apical barrier material for root-end closure in the permanent teeth of five patients.

**How to cite this article:** Gupta S, Goswami M. Use of Mineral Trioxide Aggregate in Surgical and Conventional Endodontics: A Report of Five Cases. Int J Clin Pediatr Dent 2013;6(2): 134-139.

## INTRODUCTION

The presence of healthy pulp is essential for root development and apical closure. An injury sustained between the ages of 6 and 14 can adversely affect a patient's pulpal health and interrupt or arrest root development.^1^When a severely inflamed or necrotic pulp in a tooth with an immature apex requires root canal therapy, it is difficult to accomplish due to the presence of thin, fragile walls and open apex.^2^ In these instances, apexification (root-end closure) is generally the preferred treatment. One of the treatment modality for apexification of immature teeth with severely inflamed or necrotic pulps is utilization of calcium hydroxide as an intracanal medication.^3^

While the advantage of calcium hydroxide lies in the fact that it has been widely studied and has shown success, the disadvantages are its prolonged treatment time, the need for multiple visits and radiographs.^4^ In some cases, root resorption^5^ possibly caused by trauma and increased risk of root fracture^6^ due to dressing the root canal for an extended time with calcium hydroxide have been reported in teeth undergoing apexification.

Over the last decade, mineral trioxide aggregate (MTA) has been researched extensively and reported as a possible answer to many clinical endodontic challenges.^4^ MTA offers the option of a two-visit apexification procedure, which has the benefit of better compliance and reduced number of radiographs over the multiple visit calcium hydroxide apexification, particularly in younger patients.^4^ This article presents five case reports involving use of MTA for successful management of various types of traumatic injuries to immature maxillary incisors.

## CASE REPORTS

### Case 1

**Fig. 1:**
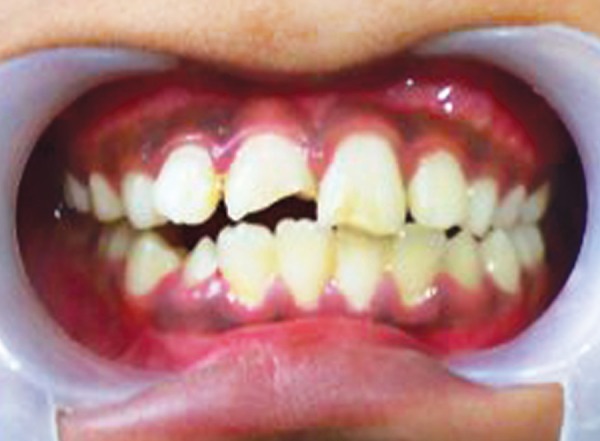
Preoperative photograph (case 1)

**Fig. 2:**
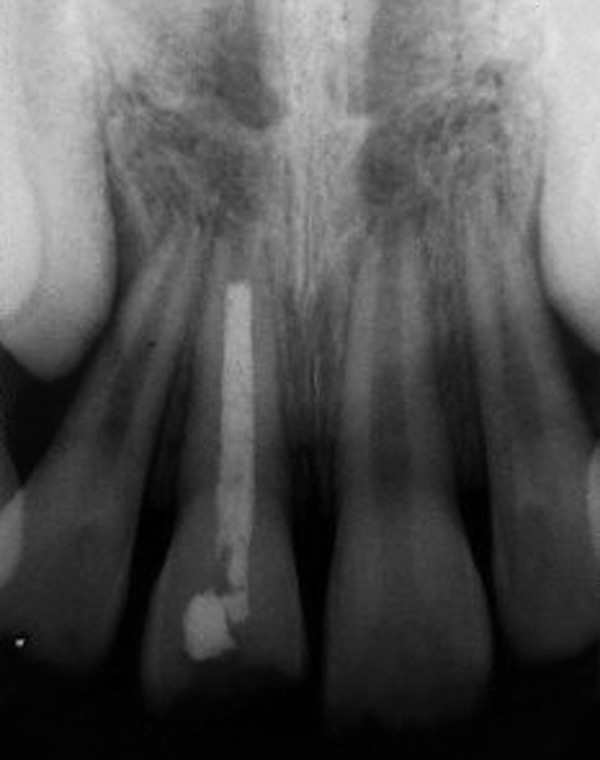
Preoperative intraoral periapical radiograph (case 1)

An 11-year-old female reported to the Department of Pediatric Dentistry with a chief complaint of unsightly appearance due to broken right upper front tooth due to trauma 2 years back. The patient gave a history of undergoing treatment from a general dental practitioner before reporting to the present department. Clinical examination revealed right maxillary central incisor with horizontal fracture at the junction of middle and coronal third of crown (Fig. 1). Patient's previous treatment records revealed some calcium hydroxide based material inserted in the root canal for apexification. Intraoral periapical radiograph revealed incomplete root end formation with the calcium hydroxide based material present in the canal but deficient near the apex (Fig. 2). The case was considered suitable for apexification using MTA.

**Fig. 3:**
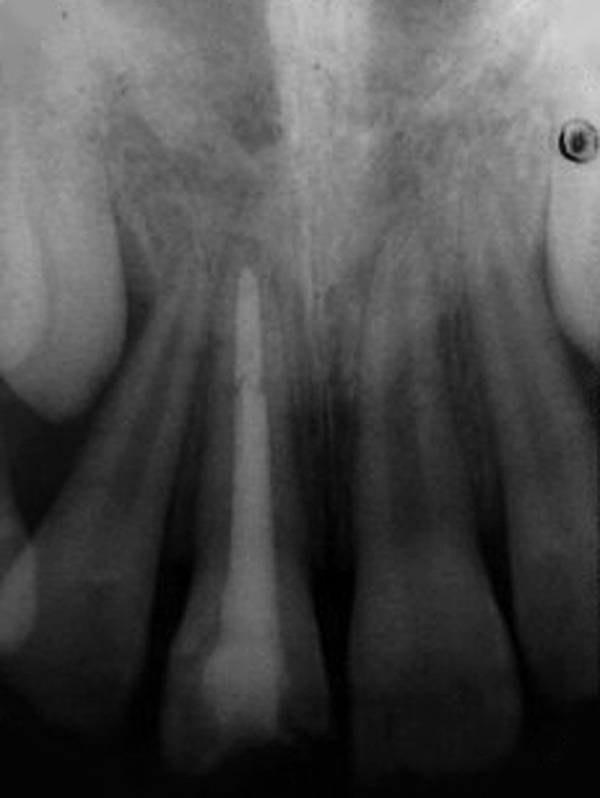
Postoperative intraoral periapical radiograph (case 1)

**Fig. 4:**
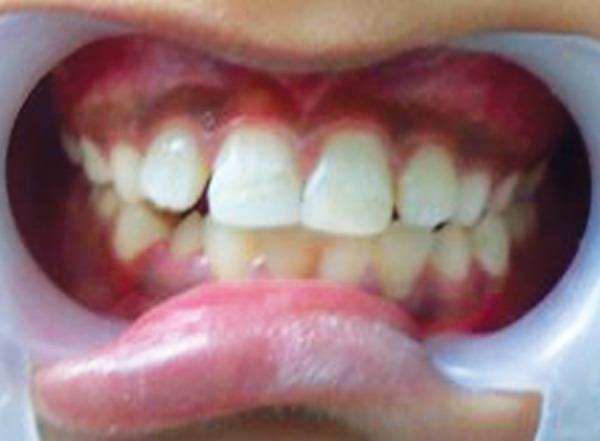
Postoperative photograph (case 1)

Obturation of the root canal was done after placement of MTA apical plug and the tooth was then restored with esthetic composite restoration (Fig. 3 and 4).

### Case 2

**Fig. 5:**
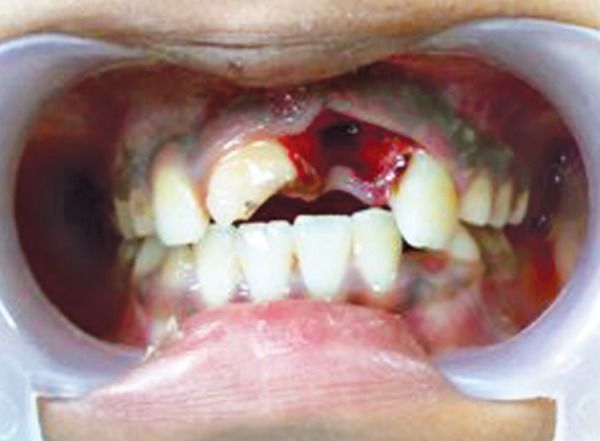
Preoperative photograph (case 2)

**Fig. 6:**
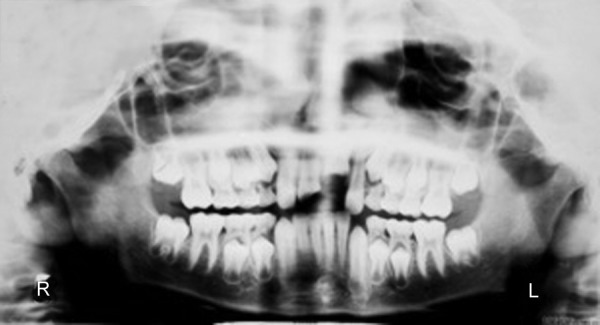
Preoperative orthopantomograph (case 2)

A 9-year-old male reported to the Department of Pediatric Dentistry with the chief complaint of a broken and a missing upper front tooth due to trauma. Clinical examination revealed oblique crown fracture of right maxillary central incisor and severe intrusion (no crown structure visible clinically) with complicated crown fracture of left maxillary central incisor (Fig. 5). All the maxillary incisors were sensitive to palpation and showed moderate mobility. Orthopantomograph confirmed the position of the intruded central incisor (Fig. 6).

**Fig. 7:**
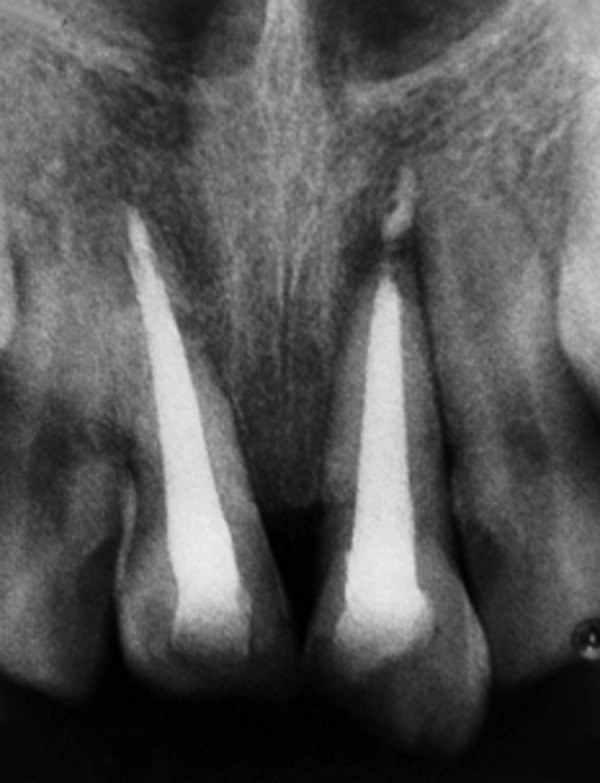
Postoperative intraoral periapical radiograph (case 2)

**Fig. 8:**
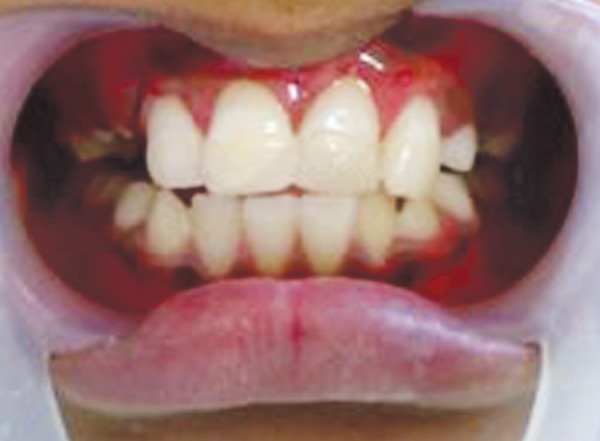
Postoperative photograph (case 2)

The intruded incisor was surgically extruded and brought into arch alignment after raising a full thickness mucoperiosteal flap. Hydroxyapatite granules were placed over the bony defect caused by associated alveolar fracture of labial cortical plate. The flap was sutured back and stabilization was achieved using arch bar for the whole maxillary arch. Suture removal and arch bar removal were done after 1 and 6 weeks respectively. Endodontic treatment of the intruded incisor was initiated and calcium hydroxide-iodoform paste (Metapex, Medicept) was placed in the root canal for a week. This was followed by placement of MTA apical plug in the next visit and obturation (Fig. 7). Endodontic treatment of the adjacent central incisor was also completed after the patient complained of pain on biting and there was persistent negative response to sensibility testing. Esthetic rehabilitation of both the teeth was done using light cured composite resin (Fig. 8).

### Case 3

**Fig. 9:**
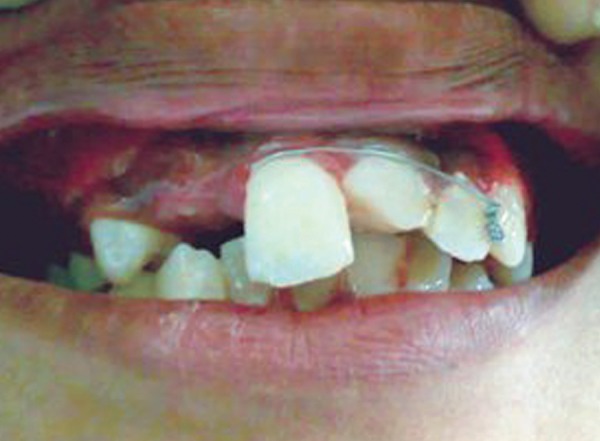
Preoperative photograph (case 3)

**Fig. 10:**
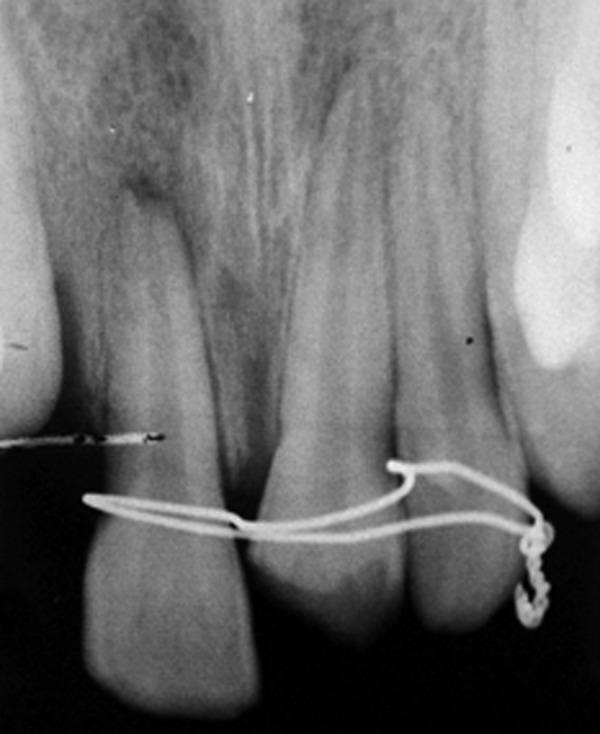
Preoperative intraoral periapical radiograph (case 3)

A 10-year-old male reported to the Department of Pediatric Dentistry with a chief complaint of loose and elongated right upper front tooth and broken left upper front tooth due to sports trauma. Clinical examination revealed extrusive luxation of right maxillary central incisor, crown fracture of left maxillary central incisor at the level of middle third and missing right maxillary lateral incisor (Fig. 9). The patient reported loss of this tooth due to trauma, which was not preserved due to lack of awareness. Both the central incisors were mobile and sensitive to percussion and palpation. Intraoral periapical radiograph revealed widening of lamina dura and periapical radiolucency and apical root resorption in relation to the extruded tooth (Fig.10). Baseline thermal testing gave negative result in the extruded tooth. Acid etch composite and wire splinting was done for two weeks to stabilize the extruded tooth.

**Fig. 11:**
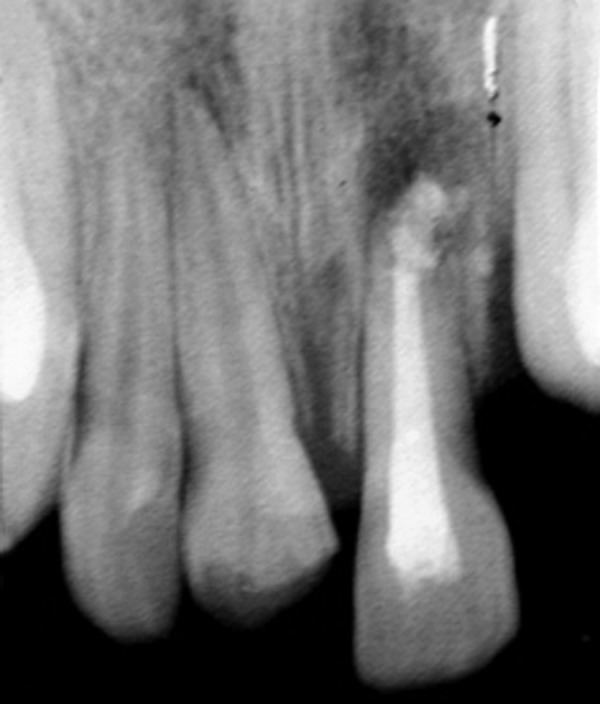
Intraoral periapical radiograph, postsurgery (case 3)

**Fig. 12:**
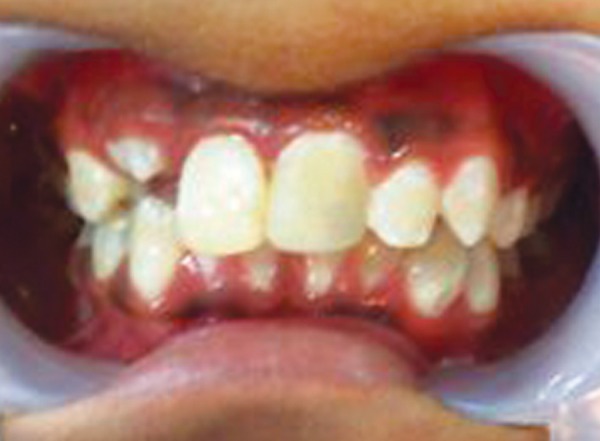
Postoperative photograph (case 3)

**Fig. 13:**
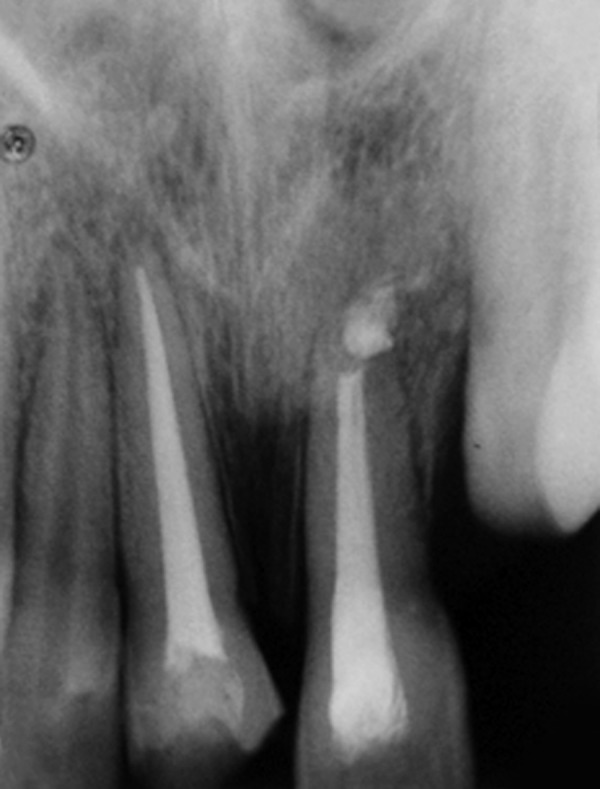
Postoperative intraoral periapical radiograph (case 3)

Three weeks later, the patient complained of spontaneous and severe pain in the region of the traumatized central incisors. It was planned to perform periapical curettage and retrograde MTA filling for this patient. For this, after obtaining adequate anesthesia, a full thickness mucoperiosteal flap was raised and periapical curettage and apicoectomy was done. MTA was packed in a retrograde manner to seal the pathways of the root canal from the periradicular tissues. The flap was replaced back and sutured. Orthograde obturation was done using gutta-percha after access opening and thorough cleaning of the root canal (Fig. 11). The tooth was stabilized using acid etch composite and wire splinting. Patient was given the necessary postoperative instructions. The sutures and splint were removed after 1 and 3 weeks, respectively, and healing was uneventful. No removable prosthesis was given for the avulsed maxillary lateral incisor as the right maxillary canine was erupting through that space. The endodontic treatment of the left maxillary central incisor was also initiated following continuous negative response to sensibility testing. Esthetic reshaping was done for the extruded tooth and composite restoration was done for the left maxillary central incisor (Fig. 12). Patient was completely asymptomatic 6 months postoperatively and the radiograph showed normal healing of the periapical area (Fig. 13).

### Case 4

**Fig. 14:**
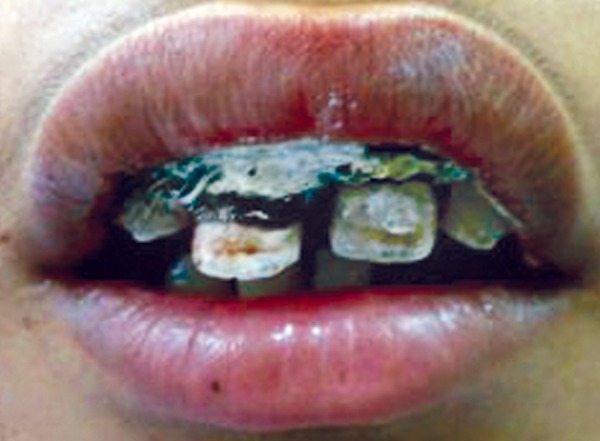
Preoperative photograph (case 4)

**Fig. 15:**
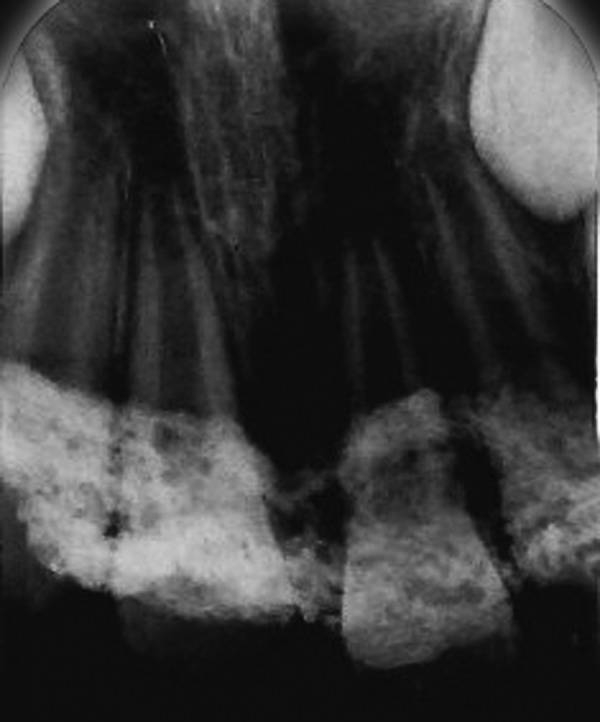
Preoperative intraoral periapical radiograph (case 4)

An 8-year-old male was referred to the Department of Pediatric Dentistry with the chief complaint of loosening of upper front teeth due to trauma. The patient gave a history of loss of his right upper front tooth which was reimplanted back into its socket about 20 minutes after trauma. Clinical examination revealed marked swelling of upper lip and extrusion of right maxillary central incisor with moderate mobility. The reimplanted tooth had been stabilized by packing gauze pieces in the labial vestibule by the referring dentist (Fig. 14). Intraoral periapical radiograph revealed marked widening of lamina dura and wide open apex of right maxillary central incisor (Fig. 15).

**Fig. 16:**
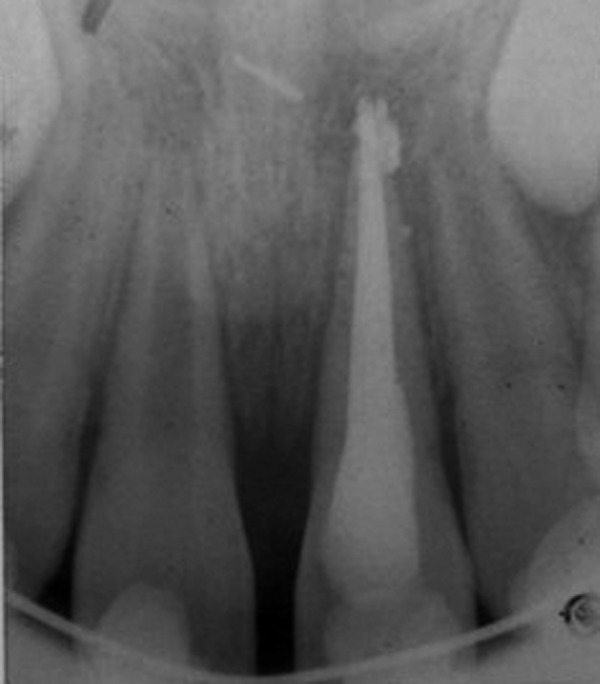
Postoperative intraoral periapical radiograph (case 4)

**Fig. 17:**
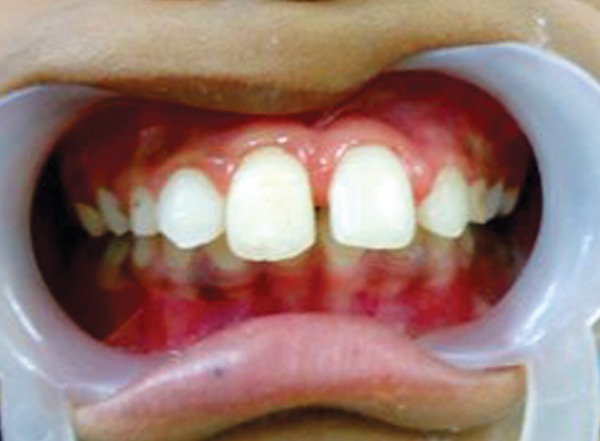
Postoperative photograph after splint removal (case 4)

Splinting was done using acid etch composite and wire for 4 weeks. Endodontic treatment of the reimplanted central incisor was initiated 1 week after splinting. The canal was filled with Metapex for 1 week for disinfection. This was followed by placement of MTA apical plug and obturation with gutta-percha (Fig. 16). The patient was recalled for splint removal after 4 weeks (Fig. 17). There was no mobility in relation to maxillary central incisors.

### Case 5

**Fig. 18:**
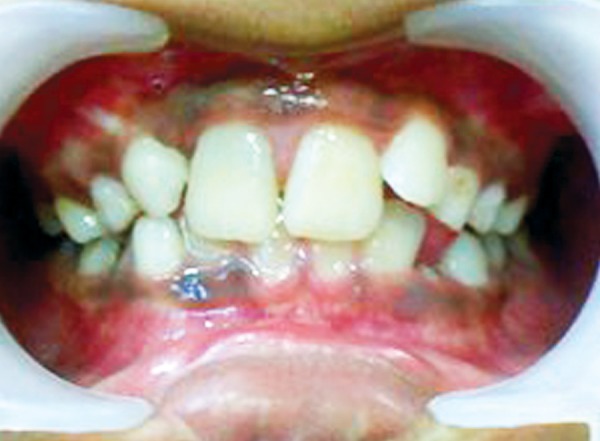
Preoperative photograph (case 5)

**Fig. 19:**
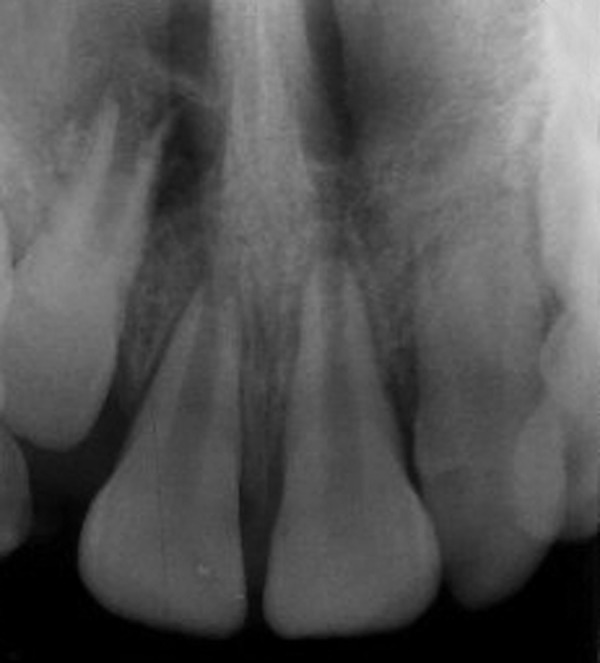
Preoperative intraoral periapical radiograph (case 5)

An 8-year-old female reported to the Department of Pediatric Dentistry with the chief complaint of pain on biting in relation to her inward displaced upper front teeth due to trauma 3 weeks back. However, there was no mobility in relation to the traumatized teeth. Clinical examination revealed the crowns of maxillary central incisors to be firm in position but palatally displaced (Fig. 18). Sensibility testing gave negative results. However, the teeth were tender to percussion. Intraoral periapical radiograph revealed widening of lamina dura and open apices in relation to both the maxillary central incisors (Fig. 19).

**Fig. 20:**
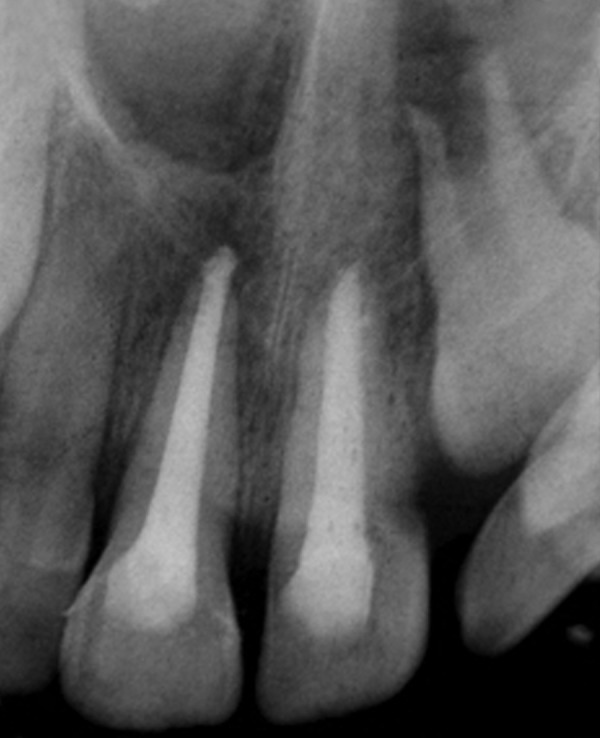
Postoperative intraoral periapical radiograph (case 5)

It was planned to perform apexification with MTA for her traumatized incisors. Access was opened for both the teeth and after thorough cleaning and debridement of the root canals; calcium hydroxide-iodoform paste (Metapex, Medicept) was inserted in the canals for a week to achieve an aseptic environment. This was followed by placement of apical MTA plugs in both the teeth and subsequent obturation using gutta-percha (Fig. 20).

In all the patients, MTA was mixed with distilled water as per the manufacturer's instructions and filled in apical third of the root canal using rear end of a size 80 gutta-percha cone. A moist cotton pellet was placed in the remaining root canal and the access opening was sealed. The patient was recalled the next day and obturation of the canal was done using gutta-percha with lateral condensation technique. All the patients have been kept on regular follow-up.

## DISCUSSION

The case 1 reports the treatment of an immature tooth initially treated with calcium hydroxide apexification technique. When the patient subsequently sought treatment for esthetic concerns, the presence of deficient calcium hydroxide filling and a defective coronal seal required revision of the endodontic procedure. The use of MTA as an apical filling material and restoration with chemically cured composite resin aid in preventing further contamination of the root canal system and restoring the esthetics. The advantages of a one-step MTA procedure were cited as reduced treatment time, reduced risk of calcium hydroxide-induced changes to dentine, and consequently reduced fracture risk, and the early placement of a sealing and possibly reinforcing coronal/intraradicular restoration.^7^

Because of the severity of intrusion and completed root development, immediate surgical repositioning of intruded incisor was planned in case 2. Arch bar was used in this case because of associated alveolar fracture and extreme mobility of the adjacent teeth. An animal *in vitro* study^8^reported that surgical repositioning of severely intruded permanent teeth with complete root development resulted in more normal orientation of the periodontal fibers and consequently less replacement resorption as the fibers are under less tension with respect to the cementum and bone walls. In addition, Andreasen et al^9^ favored surgical repositioning of moderate to severely intruded teeth with complete root development as it is potentially less time consuming, requiring fewer patient visits. Prior to use of MTA, it is necessary to remove the bacteria and their byproducts from the canal using irrigation with sodium hypochlorite and intracanal medication with calcium hydroxide for a minimum of 1 week; the latter has been demonstrated to eliminate bacteria in the root canal when applied for this period.^10^ Thus Metapex (calcium hydroxide-iodoform paste) was placed in the root canal as intracanal dressing for a week.

In case 3, as there was marked root resorption and periapical radiolucency of the extruded tooth, retrograde MTA filling was done after apicoectomy and periapical curettage. An ideal orthograde or retrograde filling material should seal the pathways of communication between the root canal system and its surrounding tissues. It should also be nontoxic, noncarcinogenic, nongenotoxic, biocompatible, insoluble in tissue fluids, and dimensionally stable. MTA was developed and recommended because existing root-end filling materials did not have the ‘ideal’ characteristics.^11^

In case 4, endodontic treatment of the avulsed tooth was initiated even though it had open apex as no attempts were made by the referring dentist to achieve disinfection of the tooth and it had not been stored in an appropriate medium prior to reimplantation. Thus, calcium hydroxide-iodoform based intracanal medicament was placed in the root canal for this purpose prior to apexification by MTA. The patient has been kept on regular follow-up to assess the outcome of the procedure.

Several authors have highlighted the unpredictable response of a tooth to pulp testing following trauma. The irregular response is caused by injury, inflammation, pressure or tension to apical nerve fibers.^12^ Development of pulp necrosis after trauma can be associated with symptoms, such as spontaneous pain or tenderness to percussion.^13^ Thus, endodontic treatment of the traumatized teeth was followed in case 5 after the patient complained of pain on biting in these teeth.

The diverse application of MTA in the practice of pediatric dentistry is evident in its use as an apical barrier in immature nonvital teeth and in the coronal fragment of fractured roots, as a pulpotomy medicament in primary and permanent teeth, a pulp-capping agent in young permanent teeth, and as a repair material for perforation and resorptive defects.^7^ Despite its many advantages, MTA has some drawbacks such as discoloration potential, presence of toxic elements in the material composition, difficult handling characteristics, long setting time, high material cost, an absence of a known solvent for this material, and the difficulty of its removal after setting.^14,15^ Although the standardized clinical approach for apexogenesis or apexification has been widely practiced, the fate of apexification as a first-line treatment for immature teeth with nonvital pulps is affected by the shifting paradigm of the management and the coming era of pulp/dentine tissue regeneration. The progress of pulp/dentine regeneration so far has been promising and is likely to work in the not so distant future.^16^
